# Visibility Versus Privacy of Physicians in the Age of Social Media

**DOI:** 10.2196/21640

**Published:** 2021-03-08

**Authors:** Sunny J Patel, Achuta K Guddati

**Affiliations:** 1 Medical College of Georgia Augusta University Augusta, GA United States

**Keywords:** social media, privacy, internet

## Abstract

As access to the internet has grown over the years, social media has become an important resource in the health care sector. Third-party physician-rating websites in particular have gained popularity. However, there are ethical implications of such websites. These websites provide a platform for patients to evaluate and review physicians and likewise increase visibility and advertisement of physicians, but they also violate the rights to privacy that these doctors should have. This paper aims to study and assess the ethical implications of these websites on the visibility and privacy of physicians. After presenting the ethical dilemma associated with such websites, it provides guidelines that can be incorporated by both physicians and third-party sites to help maintain physician privacy while providing public service in the form of advertisement and visibility.

## Introduction

Social media websites have become the new norm throughout our society. Readily available, these sites allow users to contribute, retrieve, and explore content. In the medical world, patients are increasingly relying on the internet to search for health information and inquire about health care providers [1]. Physician-rating websites in particular have grown over the years in the United States, providing patients with an avenue to influence the quality of care provided by physicians and their services. These websites come in a variety of forms and are often found in the United States as separate, third-party rating sites exclusively reviewing physicians or as hospital staff directory websites with health care–related surveys and reviews addressing patient experiences with their physicians. Many hospital systems and clinics utilize their own sources like Press-Ganey studies as internal tools [2,3], but third-party physician-rating websites have recently gained a large online presence and are the primary concern of this article. There are currently >60 third-party physician-rating websites, of which Google, Healthgrades, Vitals, Rate MD, and Yelp were noted to be among the most frequently visited [4-9]. Yelp and Healthgrades appear to be the most popular [9]. In 2010, Lagu et al [7] conducted a study in which they found that 88% of Americans used the internet to search for health-related information, out of which approximately half searched for their physicians. Over 90% of physicians are also noted to have their professional information available online, much of which is found on these websites [10]. These websites function to not only provide basic information regarding individual physicians but also enable users to enter and view reviews for specific physicians. Physician details such as personal demographic information (ie, name, address, phone, specialty, education, links to practices and professional affiliations, licenses or board certifications, insurance coverages) along with structured review questions involving care, accessibility, communication, professionalism, and billing are often provided [11]. With the growth of the internet, consumerism, and social media in recent years, further utilization of such sites has already occurred and will inevitably continue to expand [4]. Emmert et al [5] looked at the impact physician-rating websites have upon selecting a certain physician. Their study found that 65.4% and 52.2% of survey participants selected and rejected, respectively, a particular physician based on ratings shown on physician-rating websites. Similar findings of these websites swaying public opinion in regard to physician experiences were corroborated in a later investigation by Emmert et al [12] as well. In another study, physicians themselves were asked about their thoughts regarding the effect of publicizing patient opinions; 78% of surveyed physicians acknowledged that posting patient experience data publicly would negatively affect their job-related stress [13]. Suffice to say, social media has the power to shape a physician’s professional identity and experience. With the rise of online reviews and their consequences, it is pressing to understand the implications that these websites have upon physicians. While they provide a platform for advertisement and branding that can help many physicians expand their market and share their practice, there are ethical concerns regarding the privacy and visibility that such sites afford.

Yet, what exactly defines these terms: privacy and visibility? In medicine, most references to privacy deal with patient confidentiality and the Health Insurance Portability and Accountability Act of 1996. However, privacy is a convoluted term with a multitude of definitions that reference all individuals, patients, and physicians alike. In this article, privacy refers to one’s control over his or her own personal information and having the ability to limit access where he or she deems appropriate. It is an individual’s fundamental right to decide for himself or herself to what extent he or she would like to share personal details for public observation and discussion [14]. Visibility in turn is simpler to understand than privacy and refers to the attention or self-promotion one receives for advertising personal information. It primarily highlights how an individual is marketed to the public.

## Discussion

### Current Situation

Information regarding a physician is available to patients on state medical licensure websites and is provided as a service to the public by the government. Part of such information can be considered accurate and verifiable as it comes directly from state medical boards, the United States Medical Licensing Examination, and other validated sources. Given the value we as a society place on the assessment of quality of care and patient satisfaction, it is an appropriate service to the public who have a right to know about their providers. While physician-rating websites may utilize and advertise physician information, they have an inherent moral obligation to ensure that the information regarding providers listed on their websites is accurate. An ethical issue, however, arises when third-party sites aggregate information related to provider practices that may be outdated and potentially misleading to patients. Some physicians may be inaccurately classified, while others may have transitioned from their previous practice resulting in different addresses, telephone numbers, and coverage information. A simple perusal through the Better Business Bureau for complaints against third-party physician-rating websites has shown that multiple instances exist where physicians have requested that companies like Healthgrades and Vitals.com remove their personal information from their website and database for a variety of reasons, ranging from incorrect, misleading information that affects their patient population to a personal wish to no longer be publicly visible [15,16]. Even though some providers wish not to have their information publicly available or be removed from such sites, these companies and their policies require physicians to update such information themselves through their portal regardless of their opinions. However, is it an ethically acceptable practice for a company to require a physician to update his or her information when they themselves have not given consent for its dissemination? Since this information is utilized by these websites, it should be their responsibility to provide accurate information if they wish to use it and post it in a public domain. The standard argument by these companies is that the information posted on their websites is publicly available from other sources, and hence, individual consent is not required. The responsibility of accuracy of this information is sidestepped since this service is claimed to be pro bono publicito. It should also be acknowledged that vetting the accuracy of the information for each provider would be an extraordinarily onerous task. In such situations, the public expects that a reasonable amount of time and effort should have been committed to ensuring that attempts have been made to preserve accuracy of the information as much as possible. Some level of accountability should be established.

### Additional Ethical Issues

Another major ethical issue is the solicitation of reviews by third-party websites. It is an acceptable practice to invite public opinion on newsworthy issues to increase awareness and broaden the scope of discussion. But is a physician’s practice by itself newsworthy enough to solicit reviews? If this is done without the consent of the physician, then this principle could be extended to any individual whose profession deals with interaction with the public such as a cobbler, grocer, or butcher. For example, one could solicit reviews about a store at the corner of a residential block and claim it to be of public service. If the store is no more extraordinary than any other store in the neighborhood and the owners of the store have not given their consent for a third party to solicit reviews, what constitutes appropriate legal grounds to move forward to solicit reviews but not take responsibility for the accuracy of the reviews? Can such a third-party entity be held legally liable in a situation where slander and libelous material are posted online and cause subsequent damage? This issue is addressed by some websites that rate services by ensuring that the customers are indeed genuine and that their details are verifiable. Patients who decide their health care based on online reviews of health care entities should feel assured that the information being provided to them is reasonably accurate. Moreover, physicians should have the right to decide whether to allow or authorize third-party websites to broadcast their professional image and solicit reviews and evaluations on their behalf. Every individual has a reasonable expectation of privacy, and physicians are no exception. Physicians have a fundamental ethical obligation to maintaining patient confidentiality and privacy for the welfare of their patients; it becomes onerous to defend an accusation in public while strictly maintaining confidentiality. This asymmetric playing field can be addressed if patients give up their right to confidentiality should their feedback be challenged by the physician in a public forum. On one hand, broadcasting a physician’s personal information may be seen as an act of public service that empowers consumers with the opportunity to find optimal medical attention to their own personal liking. On the other hand, it can be considered an unreasonable invasion of privacy for a website to enlist a physician’s details and information—especially if the physician submitted a request in writing to remove their details from the website. A glaring example of the misuse of public trust is providing a “Thank you for your review” reply comment by physicians on these websites. If the physician did not factually provide the reply comment, then there are legal grounds for impersonation since the website falsely represented the physician for the benefit of the website company. This information is made available to others without the consent of the subject and thus violates the physician’s standards of privacy. It is ethically inconsiderate to accept unsolicited reviews on behalf of a physician who has not authorized the utilization of his or her professional information. Oftentimes, this occurs because many physicians are not even aware of their names being visible on such sites. For example, physicians of a certain area or medical group may be listed along with their phone numbers and other details gleaned through the internet. The allocation of this information can be claimed as public service but in fact, enlisting their names might invite unsolicited attention that individuals may not desire. While some physicians may feel the need to publicize and advertise their practice, others may not wish to have such attention and publicity. Privacy for these individuals serves as protection from public judgment and even more importantly, provides freedom from being consumed by constant visibility on social media—a necessary privilege that allows these individuals to control their outward appearance. That is a right that should be maintained and dictated by physicians themselves, not third-party websites unless express consent is obtained in each case.

### Summary and Future Guidelines

As the internet becomes more integral to our lives and social media expands in the United States, the emerging role of physician-rating websites and their influence cannot be ignored. These websites provide an open forum for advertisement, transparency, and feedback that may help patients make informed decisions and also improve a health care provider’s practice. However, as discussed, it presents many ethical challenges, such as the predicament of balancing the privacy and visibility of physicians. These websites encourage visibility and advertisement through self-promotion, but they also provide unsolicited attention that violates a physician’s ethical right to privacy especially when information is utilized without consent. Our hope is to maintain ethical privacy for physicians while allowing websites to provide visibility in the field in order to enhance patient and provider goals. The following list provides helpful guidelines and strategies to provide a practical solution to promoting appropriate behavior among physicians and third-party companies regarding visibility and privacy:

1. Construct the relationship between visibility and privacy as a symbiotic relationship. Create a platform or committee that focuses on creating direct, open collaboration among physicians and third-party websites to help illustrate the value of privacy and visibility. This mutual relationship may provide the foundation for more updated, claimed profiles with accurate information.
2. Educate third-party companies about the role that physician privacy and trust play in making their business reliable.
3. New residents or physicians should be contacted regarding potential advertisement of their own personal brands to ensure that their own rights of privacy as well as the accuracy of physician information are not violated. Follow-up should be designated annually to maintain validity. If the physician is unavailable to provide permission, it is appropriate to utilize information found on state-accredited sites, but a medium should be used to make physicians aware that information is being used. In such a case, public service to the community is provided without violating privacy rights as steps have been taken to communicate with physician.
4. Respect a request of privacy from a physician to remove details about their profile. It should be noted that such removal should not be selective to only negative reviews. Giving up presence on a website means giving up both good and bad reviews.
5. Third-party websites should take responsibility for any civil or criminal liability stemming from damages sustained by physicians due to false information on their websites. Even if the website only hosted the information, by providing a platform to disseminate unverified and false information, they have become an accomplice in a wrongdoing.

Both providers and third-party companies should take an active role in the development of quality physician-rating websites that ensure an appropriate level of visibility while maintaining a physician’s ethical right to privacy. Joint collaboration will not only result in optimal quality and accuracy of updated information but also lead to a more satisfied population of providers and patients alike. If a physician requests privacy, it should be respected barring exceptional cases of newsworthiness.
